# Review of *Anhoplocampa* Wei (Hymenoptera, Tenthredinidae), with description of a new species and a new combination

**DOI:** 10.3897/zookeys.159.2476

**Published:** 2011-12-23

**Authors:** Meicai Wei, Gengyun Niu

**Affiliations:** 1 College of Life Science and Technology, Central South University of Forestry and Technology, 498 South Shaoshan Road, Changsha 410004, P. R. China

**Keywords:** Hymenoptera, Tenthredinidae, *Anhoplocampa*, new species, new combination, China

## Abstract

*Anhoplocampa* is redescribed based on new material. *Anhoplocampa bicoloricornis* **sp. n.** from China is described. *Anhoplocampa yunanensis* (Haris & Roller, 1999), **comb. n.** is transferred from *Trichiocampus*. A key to species of *Anhoplocampa* is provided. The differences between *Anhoplocampa* and *Trichiocampus* Hartig, 1837, *Priophorus* Dahlbom, 1835, *Hoplocampa* Hartig, 1837 and *Renonerva* Wei & Nie, 1998 are briefly discussed.

## Introduction

Wei (1998) described *Anhoplocampa* and its type species *Anhoplocampa fumosa* from China based on a single female. The genus is quite peculiar from other taxa of Nematinae by with the presence of a distinct smoky band below pterostigma in the forewing; mesepisternum with a deep, broad and strongly curved furrow along the anterior and dorsal margins of the mesepisternum, and the epicnemium long, narrow, and strongly elevated.

Specimens of *Anhoplocampa* are rarely collected in the field. Except for the holotype of the type species, which was collected from Sichuan Province in 1983, one specimen was collected in Henan Province in 2001 and one in Yunnan Province in 2004. The two specimens represent two additional species of the genus.

In 1999, Haris and Roller described *Trichiocampus yunanensis* from Yunnan, China based on a female specimen. Examination of the holotype shows that it is a species of *Anhoplocampa* and identical to the specimen collected from Yunnan in 2004.

*Anhoplocampa* is redescribed based on new material. A new species is described and a new combination is proposed below.

## Material and methods

Terminology of sawfly genitalia follows Ross (1945). Wing venation is shown in Fig. 7 with names of cells on the right side and names of veins on the left side.

The images were obtained using a Nikon D2x digital camera and Motic BA400 microscope and further processed with Helicon Focus 5.1(©HeliconSoft) and Adobe Photoshop CS2 software.

Abbreviations used are: OOL = distance between the eye and outer edge of lateral ocellus; POL = distance between the mesal edges of the lateral ocelli; OCL = distance between a lateral ocellus and the occipital carina or hind margin of the head.

Rules for spelling Chinese personal and place names follow GB/T 16159-1996 and ISO 7098: 1991: “Chinese people’s names are to be written separately with the surname first, followed by the personal name written as one word, with the initial letters of both capitalized.”. “Chinese place names should be alphabetized according to the “Spelling Rules for Chinese Geographical Place Names,” document no. 17 (1984) of the State Committee on Chinese Geographical Place Names.”

Specimens examined are deposited in the Insect Collection of the Central South University of Forestry and Technology, Changsha, P. R. China (CSCS) and the Hungarian Natural History Museum, Budapest, Hungary (HNHM).

## Taxonomy

### Anhoplocampa

Wei, 1998

http://species-id.net/wiki/Anhoplocampa

Anhoplocampa  Wei, 1998: 14. Type species: *Anhoplocampa fumosa* Wei, 1998, by original designation.

#### Description.

**Figures 1–6.**
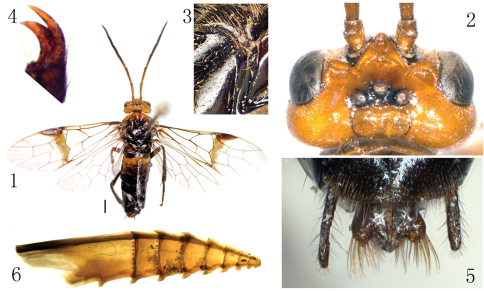
*Anhoplocampa bicoloricornis* sp. n., holotype **1** Adult female, dorsal view (scale bar = 1mm) **2** Head, dorsal view **3** Prepectus, epicnemium and dorsal part of mesepisternum **4** Claw **5** Cerci and apical sheath, dorsal view **6** Lancet

**Figures 7–13.**
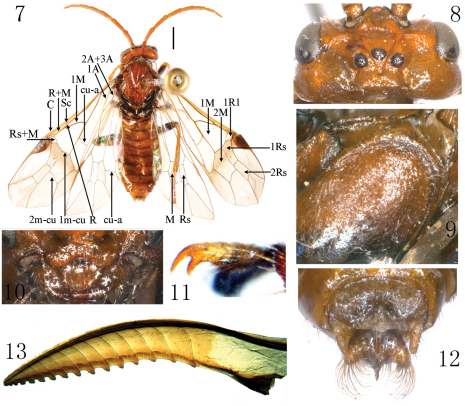
*Anhoplocampa yunanensis* (Haris & Roller, 1999) comb. n., specimen from Xiaozhongdian, Yunnan **7** Adult female, dorsal view (scale bar = 1mm) **8** Head, dorsal view **9** Prepectus, epicnemium and dorsal part of mesepisternum **10** Clypeus **11** Claw **12** Cerci and apical sheath, dorsal view **13** Lancet

**Figures 14–18.**
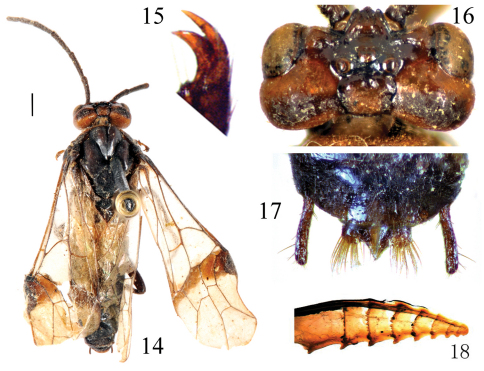
*Anhoplocampa fumosa* Wei, 1998, holotype **14** Adult female, dorsal view (scale bar = 1mm) **15** Claw **16** Head, dorsal view **17** Cerci and apical sheath, dorsal view **18** Lancet

Body length 7–11 mm. Clypeus flat, anterior margin emarginated (Fig. 10); malar space about as long as diameter of lateral ocellus; eyes small, distance between eyes at level of antennal sockets (toruli) 1.2–1.8 × greatest diameter of eye; supraantennal area distinctly protruding between antennal sockets; frontal area surrounded by strong carinae (Figs 2, 8, 16), upper margin of lateral fovea carinate; occipital carina absent; left mandible in lateral view with swollen base, narrowing to thin blade-like apex (similar to Benson 1958, fig. 380). Head weakly dilated behind eyes in dorsal view (Figs 2, 8, 16). Antenna long and slender, basal two antennomeres short, much broader than long, third antennomere clearly shorter than fourth antennomere, other flagellomeres subequal in length. Prepectus lanceolate, distinct, about 3 × longer than wide (Fig. 3); epicnemium narrow, strongly elevated, furrow between epicnemium and mesepisternum deep and broad in entire length, strongly curved in upper part across upper 0.10 of mesepisternum (Figs 3, 9). Inner tibial spur of front leg bifid apically, much longer than outer spur. Hind tibia with longitudinal furrow on outer side, tibial spurs short, 1.1–1.3 × apical breadth of tibia; hind tarsus about 0.8 × length of hind tibia; metabasitarsus about 1.1 × length of following 3 tarsomeres together; claw without basal lobe, inner tooth large, slightly shorter than or about as long as outer tooth (Figs 4, 11, 15). Forewing with a distinct dark band below pterostigma, vein R+M longer than cu-a, Sc slightly basad apex of vein 1M; vein 2r1 absent; vein 1M strongly convergent toward pterostigma with 1m-cu; cell 2Rs subequal to 1Rs in length and about 2 × as long as broad; 2m-cu joining cell 2Rs at basal 0.20–0.25; cell 1R1 about as long as broad, cell 2M longer than broad; vein 2A+3A meeting 1A at basal 0.3, basal anal cell closed; cu-a meeting cell 1M at about middle to basal 0.4 (Figs 1, 7, 14). Hind wing with cells Rs and M closed, anal cell closed, petiole of anal cell as long as width of anal cell and subequal to vein cu-a (Figs 1, 7). Cerci usually slender (Figs 5, 12, 17). Ovipositor sheath not longer than middle tibia, apical section of sheath with 3 processes in dorsal view, scopae distinct (Figs 5, 12, 17). Lancet with or without ctenidia, without stout annular setae, serrulae without denticles (Figs 6, 13, 18).

#### Distribution.

China (Yunnan, Sichuan, Henan).

#### Remarks.

*Anhoplocampa* Wei, 1998 is similar to *Trichiocampus* Hartig, 1837 and *Priophorus* Dahlbom, 1835 but differs from those two genera by the very narrow and strongly elevated epicnemium; presence of a broad, deep furrow between the epicnemium and mesepisternum, strongly curved in the upper part of the mesepisternum; prepectus distinct; front wall and upper margin of the lateral fovea strongly carinate; left mandible in lateral view with a swollen base and a thin blade-like apex; vein R+M in forewing longer than cu-a; apical sheath with large scopae; cerci long and slender; forewing with a dark band below pterostigma; petiole of hind anal cell as long as width of anal cell; as well, in *Trichiocampus* and other Cladiini the vein 1M meets vein R close to the point where Rs+M meets R+M; vein 1M is far removed from that point in *Anhoplocampa*.

*Anhoplocampa* differs from *Hoplocampa* Hartig, 1837 by the much larger body; antenna longer than the abdomen with the scape and pedicel much broader than long; forewing with a dark band below pterostigma and vein 2r absent; petiole of anal cell of hind wing not longer than cu-a; left mandible in lateral view with a swollen base and a thin blade-like apex; epicnemium very narrow and strongly elevated, with a broad and deep furrow between the epicnemium and mesepisternum, strongly curved in the upper part of the mesepisternum; supraclypeal area strongly protruding between antennal sockets and distance between antennal sockets distinctly narrower than the inner orbit at the same level; frontal walls strongly carinate; and ovipositor sheath shorter than middle tibia and with distinct scopae.

*Anhoplocampa* is also somewhat similar to *Renonerva* Wei & Nie (Wei and Nie 1998) sharing the narrow and strongly elevated epicnemium with a deep and broad furrow between epicnemium and mesepisternum, but *Anhoplocampa* differs from the latter by the robust body and stout antenna; the distinctly emarginated clypeus; malar space about as long as diameter of middle ocellus; prepectus distinct; cerci not linear; forewing with a dark band below pterostigma, vein 2r1 absent, 2A+3A on forewing straight and the basal anal cell open; the hind basitarsus longer than the following three tarsomeres together; ovipositor sheath with distinct scopae; lancet not strongly reduced and lamnium not shorter than radix.

*Anhoplocampa* differs from *Hemichroa* Stephens, 1835 in the very narrow and strongly elevated epicnemium; presence of a broad, deep furrow between the epicnemium and mesepisternum, strongly curved in the upper part of the mesepisternum; front wall and upper margin of the lateral fovea strongly carinate; left mandible in lateral view with a swollen base and a thin blade-like apex; apical sheath with large scopae; forewing with a dark band below pterostigma, vein 2r1 absent and the middle petiole of anal cell on forewing shorter than vein R+M.

*Anhoplocampa* also shares some characters with *Pristiphora* Latreille, 1810, for example the bladelike mandibles and the apical sheath with distinct scopae. But *Anhoplocampa* differs from *Pristiphora* by the very narrow and strongly elevated epicnemium; presence of a broad, deep furrow between the epicnemium and mesepisternum; front wall and upper margin of the lateral fovea strongly carinate; the forewing with a dark band below pterostigma, the vein 2A+3A curved up and meeting 1A and therefore the basal anal cell closed.

Three species of *Anhoplocampa* are now known. They can be identified with the following key.

##### Key to species of Anhoplocampa Wei

**Table:** 

1	Clypeus deeply incised to about 0.5 × length of clypeus (Fig. 10); postocellar area and temple flat (Fig. 8); cerci not extending to end of sheath in dorsal view (Fig. 12); forewing with a not strongly defined cross band posterior of pterostigma (Fig. 7); head, antenna, mesothorax dorsally, abdomen entirely, and all tibiae and tarsi reddish brown; cu-a of fore wing meeting cell 1M at about middle (Fig. 7); upper half of mesepisternum distinctly microsculptured (Fig. 9); lateral furrows of postocellar area deep and broad (Fig. 8); lancet slender, with 16 serrulae and 14 annuli, annuli without ctenidia (Fig 13). Yunnan	*Anhoplocampa yunanensis*
–	Clypeus shallowly incised to about 0.25 × length of clypeus; postocellar area declined posteriorly, temple distinctly convex (Figs 2, 16); apex of cercus extending clearly beyond apex of sheath in dorsal view (Figs 5, 17); cross band posterior of pterostigma dark, sharply defined (Figs 1, 14); at least apical half of antenna, most of mesothorax dorsally, abdomen except 2^nd^ tergite, hind tibia and tarsus entirely black; cu-a of fore wing meeting cell 1M at about basal 0.4 (Figs 1, 14); mesepisternum strongly shiny, without microsculpture (Fig. 3); lateral furrows of postocellar area very fine or narrow (Figs 2, 16); lancet short, with 9 serrulae and 8 annuli, annuli with ctenidia (Figs 6, 18)	2
2	Antenna entirely black (Fig. 14); labrum, supraclypeal area and frons black; prescutum entirely black; middle tibia and middle tarsus pale brown; posterior half of postocellar area strongly declined (Fig. 16); frontal basin longer than broad, deep, 0.5 × as deep as width of basin; distance between eyes at level of antennal sockets 1.5 × wider than greater diameter of eye; anterior part of the deep brown band on forewing almost as broad as length of pterostigma (Fig. 14); lancet with teeth of 1^st^ ctenidium obtuse (Fig. 18). Sichuan	*Anhoplocampa fumosa*
–	Antenna with basal half reddish brown, apical half black (Fig. 1); head orange except for obscure dark band across postocellar area (Fig. 2); anterior 0.6 of prescutum reddish brown (Fig. 1); middle tibia and tarsus black brown; posterior half of postocellar area weakly declined; frontal basin broader than long, very shallow, 0.13 × as deep as width of basin (Fig. 2); distance between eyes at level of antennal sockets 1.2 × wider than greater diameter of eye; anterior part of the deep brown band on forewing about as broad as 0.5 × length of pterostigma (Fig. 1); lancet with teeth of 1^st^ ctenidium acute (Fig. 6). Henan	*Anhoplocampa bicoloricornis*

### Anhoplocampa bicoloricornis sp. n.

urn:lsid:zoobank.org:act:792A18AE-754C-4F1C-9685-BB411A50FA24

http://species-id.net/wiki/Anhoplocampa_bicoloricornis

Figs 1–6 

#### Description.

**Female** (holotype, Fig. 1). Body length 9 mm (excluding antenna, cerci and sheath). Head orange, extreme narrow margin of antennal socket and a transverse band over ocelli black, apical half of antenna black; thorax and abdomen black, pronotum except lower corners, anterior 0.6 of prescutum and anterior corner of lateral lobe of scutum orange; 2^nd^ abdominal tergite entirely and a spot near spiracle of 3^rd^ tergite pale brown; legs black, tibia and tarsus of fore leg yellow brown. Wings hyaline, vein C except for both ends pale brown, other veins and pterostigma black brown, anterior breadth of sharply defined smoky macula on forewing about half length of pterostigma. Body hairs brown.

Body shiny, without distinct punctures and microsculptures. Clypeus shallowly and roundly emarginated to about 0.25 length of clypeus; malar space slightly longer than diameter of lateral ocellus; postocellar area 2 × as broad as long, with a shallow and broad middle furrow, posterior half of postocellar area weakly declined; postocellar furrow distinct; lateral furrows very shallow, weakly curved; temple weakly bulged; frontal basin shallow, 1.3 × broader than long, with depth about 0.13 × breadth of basin; frontal walls broad, strongly elevated (Fig. 2); distance between eyes at level of antennal sockets 1.2 × wider than greater diameter of eye. Antenna slender, as long as body, slightly tapering towards apex (Fig. 1). Prepectus, epicnemium and dorsal part of mesepisternum as Fig. 3. Claw with inner tooth hardly shorter and distinctly broader than outer tooth (Fig. 4). Forewing: cell 2Rs slightly longer than 1Rs, vein cu-a joining cell 1M at basal 0.43. Cerci and apical sheath in dorsal view as Fig. 5, cerci about 5.5 × as long as broad. Lancet with 8 annuli and 9 serrulae, 1^st^ annulus with distinct annular teeth (Fig. 6).

**Male.** Unknown.

#### Distribution.

China (Henan Province).

#### Etymology.

The new species is named after the color of the antenna.

#### Holotype.

♀, China: Henan Province, Songxian, Baiyunshan, 1800 m, 2001.VI.2, Zhong Yihai leg. (CSCS).

#### Remarks.

See above key for differences between the three species of the genus.

### Anhoplocampa yunanensis

(Haris & Roller, 1999) comb. n.

http://species-id.net/wiki/Anhoplocampa_yunanensis

Figs 7–13

Trichiocampus yunanensis  Haris & Roller, 1999: 231–232.

#### Description.

**Female** (Fig. 7). Body length 7 mm (excluding antenna, cerci and sheath). Body orange, extreme narrow margin of antennal socket, propleuron, parapsis of mesothorax largely, narrow posterior margin of mesoscutellum, parapsis of metanotum, posterior of metascutellum, ventral half of mesopleuron, ventral margin of metapleuron, black; legs black, apical 1/3 of fore femur, all tibiae and tarsi yellow brown. Wings hyaline, apical third weakly infuscate, vein C pale brown, other veins and pterostigma black brown, anterior breadth of a feebly defined smoky macula about half length of pterostigma. Body hairs silver brown.

Body shiny, dorsal side of pronotum distinctly punctured, dorsal half of mesopleuron distinctly microsculptured (Fig. 9). Clypeus distinctly and roundly emarginated to about half length of clypeus (Fig. 10); malar space about 1.15 × diameter of lateral ocellus; postocellar area flat, 2.2 × as broad as long, with a deep middle fovea and a shallow middle furrow, posterior of postocellar area not declined; postocellar furrow broad and shallow; lateral furrows deep and very broad, weakly divergent backwards; temple flat; frontal basin shallow, 1.1 × as broad as long, with depth about 0.13 × breadth of basin; frontal walls distinctly elevated, not very sharp (Fig. 8); distance between eyes at level of antennal sockets 1.8 × wider than greater diameter of eye. Antenna stout, 1.2 × length of abdomen, much shorter than thorax and abdomen together, tapering towards apex (Fig. 7). Prepectus, epicnemium and dorsal part of mesepisternum as Fig. 9. Claw with inner tooth clearly shorter and slightly broader than outer tooth (Fig. 11). Forewing: cell 2Rs slightly shorter than 1Rs, vein cu-a joining cell 1M at middle. Cerci and apical sheath in dorsal view as Fig. 12, cerci about 3.5 × as long as broad, not beyond apex of sheath. Lancet slender, with 14 annuli and 16 serrulae, annuli simple, without annular teeth (Fig. 13).

**Male.** Unknown.

#### Distribution.

China (Yunnan).

#### Specimens examined.

1♀, holotype: “China, Yunan [Yunnan], 24-29. VI. 93, 50 km N. of Lijieng [Lijiang], Yulongshan, Nat. Res., E. Jednek, O. Sausa leg.” (HNHM); 1 female, “Xiaozhongdian, Shangri-La, Yunnan, China, alt. 3000m, 19 July 2004, Xiao Wei coll.” (CSCS).

#### Remarks.

This species is easily separated from the other *Anhoplocampa* species by the orange body; antenna stout and much shorter than body; forewing with a weak cross band below pterostigma; postocellar area not declined and with deep and broad lateral furrows; the upper half of mesopleuron distinctly microsculptured; cerci shorter; clypeus deeply incised; claw with inner tooth clearly shorter than outer tooth; cu-a of fore wing meeting cell 1M at about middle; and female lancet slender with 14 simple annuli.

Haris and Roller (1999) placed this species in *Trichiocampus*. However, left mandible in lateral view bearing a strongly swollen base and narrowing to a thin blade-like apex, the long vein R+M, the narrow and strongly elevated epicnemium with a deep and strongly curved furrow, and the distinctly carinate frontal walls show that it is member of *Anhoplocampa* and does not belong to *Trichiocampus*.

The weak cross-band of the forewing, the comparatively short antenna, the short cerci, the slender lancet with simple annuli, the flat temple and postocellar area of *Anhoplocampa yunanensis* show that it is morphologically remote from *Anhoplocampa bicoloricornis* and *Anhoplocampa fumosa*.

### Anhoplocampa fumosa

Wei, 1998

http://species-id.net/wiki/Anhoplocampa_fumosa

Figs 14–18

Anhoplocampa fumosa  Wei, 1998: 15.

#### Description.

**Female** (holotype, Fig. 14). Body length 11 mm (excluding antenna, cerci and sheath). Head dark orange, narrow margin of antennal socket, frons and ocellar area, a broad transverse band along posterior margin of head black; antenna entirely black; thorax and abdomen black, middle half of pronotum orange; 2^nd^ abdominal tergite largely pale brown; legs black, fore tibia and tarsus yellow brown, middle tibia and tarsus pale brown. Wings hyaline, apical third of forewing weakly infuscate, veins and pterostigma black brown, anterior breadth of a sharply defined smoky band below pterostigma about as long as pterostigma. Body hairs brown.

Body shiny, without distinct punctures and microsculptures. Clypeus shallowly and roundly emarginated to about 0.25 × length of clypeus; malar space slightly longer than diameter of lateral ocellus; postocellar area 2 × as broad as long, with a shallow middle fovea, posterior half of postocellar area strongly declined; postocellar furrow distinct; lateral furrows shallow but distinct, weakly curved; temple distinctly bulged; frontal basin long and deep, 1.2 × longer than broad, with depth about 0.5 × breadth of basin; frontal walls broad, strongly elevated (Fig. 16); distance between eyes at level of antennal sockets 1.5 × wider than greater diameter of eye. Antenna slender, 1.3 × length of abdomen, not tapering towards apex (Fig. 14). Prepectus, epicnemium and dorsal part of mesepisternum similar to Fig. 3. Claw with inner tooth hardly shorter and distinctly broader than outer tooth (Fig. 15). Forewing: cell 2Rs slightly longer than 1Rs, vein cu-a joining cell 1M at basal 0.4. Cerci and apical sheath in dorsal view as Fig. 17, cerci about 5.5 × as long as broad, middle tooth of sheath acute and much longer than roundish lateral teeth. Lancet with 8 annuli and 9 serrulae, 1^st^ annulus with obscure annular teeth (Fig. 18).

**Male.** Unknown.

#### Distribution.

China (Sichuan Province).

#### Specimens examined.

1♀, holotype: “China, Sichuan, Baoxing, 2200 m, 27 June, 1983, Xiong Jiang leg.” (CSCS).

## Supplementary Material

XML Treatment for Anhoplocampa

XML Treatment for Anhoplocampa bicoloricornis

XML Treatment for Anhoplocampa yunanensis

XML Treatment for Anhoplocampa fumosa
